# High-Sensitivity Cardiac Troponin I in Apparently Healthy Blood Donors: Cross-Sectional Distribution and Discordance with Conventional Cardiovascular Risk Assessment Methods

**DOI:** 10.3390/diagnostics16142203

**Published:** 2026-07-15

**Authors:** Stefano Pignalosa, Valeria Carnazzo, Sara Tartaglione, Massimo Pieri, Raffaella Marzano, Bruno Daniele Leoni, Francesco Versaci, Mariapaola Marino, Umberto Basile, Francesco Equitani

**Affiliations:** 1 Department of Clinical Pathology, Santa Maria Goretti Hospital, 04100 Latina, Italy; s.pignalosa@ausl.latina.it (S.P.); v.carnazzo@ausl.latina.it (V.C.); s.tartaglione@ausl.latina.it (S.T.); u.basile@ausl.latina.it (U.B.); 2 Unit of Laboratory Medicine, University Hospital Tor Vergata, 00133 Rome, Italy; massimo.pieri@uniroma2.it; 3 Department of Experimental Medicine, University of Rome Tor Vergata, 00133 Rome, Italy; 4 Operating Unit Transfusion Center, Santa Maria Goretti Hospital, 04100 Latina, Italy; r.marzano@ausl.latina.it (R.M.); f.equitani@ausl.latina.it (F.E.); 5 Abbott Core Diagnostics Italy, 00144 Rome, Italy; bruno.daniele.leoni@gmail.com; 6 Division of Cardiology, Santa Maria Goretti Hospital, 04100 Latina, Italy; f.versaci@ausl.latina.it; 7 Dipartimento di Medicina e Chirurgia Traslazionale, Sezione di Patologia Generale, Università Cattolica del Sacro Cuore, 00168 Rome, Italy; 8 Fondazione Policlinico Universitario “A. Gemelli” I.R.C.C.S., 00168 Rome, Italy

**Keywords:** high-sensitivity cardiac troponin I, blood donors, cardiovascular risk assessment, biomarker categorization, Framingham Risk Score, SCORE2, cross-sectional study

## Abstract

**Background**: Interpreting low-grade of high-sensitivity cardiac troponin I (hs-TnI) concentrations reflecting myocardial injury in asymptomatic populations remains clinically uncertain. This study characterized predefined sex-specific hs-TnI categories in apparently healthy blood donors and compared them with conventional cardiovascular assessment methods. **Methods**: We performed a cross-sectional analysis of 292 blood donors (77 women, 26.4%; 215 men, 73.6%; age range, 40–65 years). Hs-TnI was measured using the Abbott ARCHITECT STAT High Sensitive Troponin-I assay and categorized according to predefined sex-specific thresholds. Categories were compared with clinical variables, cholesterol/HDL-ratio categories, Framingham Risk Score categories, and SCORE2 in eligible participants (*n* = 287). Additional analyses included non-parametric comparisons, Spearman correlations, agreement statistics, and multivariable models. A sensitivity analysis used sex-specific empirical hs-TnI tertiles, and effect sizes with bootstrap 95% confidence intervals were reported for key comparisons. **Results**: Hs-TnI categories were low in 253 subjects (86.6%), intermediate in 17 (5.8%), and elevated in 22 (7.5%). Hs-TnI was markedly skewed, especially in the elevated category (median, 21.5 ng/L; IQR, 15.25–36.0; range, 11–199). Agreement with conventional categories was low, with weighted kappa values of 0.035 versus Framingham, −0.008 versus cholesterol/HDL-ratio categories, and 0.076 versus SCORE2. Conventional cardiovascular variables showed limited independent association with intermediate/elevated hs-TnI categories. Tertile-based sensitivity analyses yielded weighted kappa values of 0.045 versus Framingham, 0.033 versus cholesterol/HDL-ratio categories, and 0.037 versus SCORE2. **Conclusions**: In selected blood donors, predefined hs-TnI categories showed limited concordance with conventional cardiovascular scores, exploratory-wise suggesting a distinct phenotype of low-grade myocardial injury.

## 1. Introduction

Cardiovascular diseases remain a leading cause of morbidity and mortality worldwide. In primary prevention, cardiovascular risk assessment is usually based on multivariable prediction models that combine demographic features with conventional risk factors, including lipid profile, blood pressure, smoking status, and diabetes [1,2,3,4,5,6].

High-sensitivity cardiac troponin assays now allow very low circulating troponin concentrations to be quantified in many apparently healthy individuals [7,8,9,10]. In population-based cohorts and meta-analyses, higher hs-TnI concentrations have been associated with incident cardiovascular events, heart failure, and cardiovascular mortality, even below the 99th percentile and after adjustment for conventional risk factors [11,12,13,14,15,16,17,18]. Recent expert reviews have therefore proposed hs-TnI as a candidate adjunctive biomarker for cardiovascular risk refinement in asymptomatic individuals, while emphasizing the need for careful clinical pathways and prospective validation [17,18].

The possible integration of hs-TnI into cardiovascular prevention therefore requires a clear distinction between analytical validity, clinical validity, and clinical utility [18,19,20,21,22,23]. Cross-sectional studies can support biological plausibility and describe biomarker phenotypes, but they cannot establish prognostic reclassification, outcome benefit, or clinical implementation by themselves.

Blood donors represent a selected group of apparently healthy individuals who undergo standardized medical screening before donation. This setting offers a pragmatic opportunity to examine hs-TnI distribution in a low-apparent-risk population, while acknowledging that donor selection limits generalizability. The primary objective of this study was to compare the distribution of predefined sex-specific hs-TnI categories with conventional cardiovascular assessment methods in apparently healthy blood donors. Secondary objectives were to evaluate clinical correlates of hs-TnI categories, assess sex-specific patterns, and quantify concordance or discordance between hs-TnI categories, cholesterol/HDL-ratio categories, and Framingham Risk Score categories. The contribution of this study is not the validation of hs-TnI as a prognostic screening tool. Rather, it shows that predefined hs-TnI categories identify a small biomarker-defined subgroup within an apparently healthy donor population and overlap only modestly with Framingham, cholesterol/HDL-ratio categories, and SCORE2. This supports the hypothesis that hs-TnI may reflect a cardiac-specific injury phenotype that complements, but does not replace, conventional cardiovascular assessment.

## 2. Materials and Methods

### 2.1. Study Design, Population and Clinical Assessment

This observational, cross-sectional study included voluntary blood donors enrolled over a 12-month period at the Transfusion Center of Santa Maria Goretti Hospital, Latina, Italy. Participants were considered apparently healthy at the time of donation according to routine donor eligibility procedures, including medical history, physical examination, and standard screening laboratory tests. The cohort was therefore interpreted as a selected healthy-donor population, not as a representative sample of the general population.

The analytical cohort included donors who passed routine donation eligibility and had the variables required for the present analysis. No additional study-specific exclusion was applied solely on the basis of overweight or obesity, mild dyslipidemia, borderline blood pressure, smoking, or prediabetic-range glucose values. Accordingly, 186 of 292 participants (63.7%) were pre-obese or obese, 56 (19.2%) had hypertension or borderline blood pressure, 72 (24.7%) were current smokers, and 22 (7.5%) had a prediabetes/diabetes classification. The analytical dataset did not distinguish first-time from repeat donors; therefore, stratification by donation history was not possible. In this study, “apparently healthy” refers to eligibility for blood donation at the index visit rather than to the complete absence of cardiovascular risk factors.

Available clinical variables included age, sex, body mass index, smoking status, systolic blood pressure and blood pressure status, fasting glucose/diabetes status, lipid profile, creatinine, C-reactive protein, family history, and Framingham Risk Score. Medication use, physical activity, electrocardiographic findings, echocardiographic data, NT-proBNP, ApoB, Lipoprotein(a), and HbA1c were not available.

The study was conducted in accordance with the Declaration of Helsinki and local ethical regulations. Data were anonymized before analysis.

### 2.2. Hs-TnI Measurement and Predefined Categories

Hs-TnI concentrations were measured using the Abbott ARCHITECT STAT High Sensitive Troponin-I assay. The assay fulfills accepted analytical criteria for high-sensitivity cardiac troponin testing, including imprecision of ≤10% at the 99th percentile and measurable concentrations in a substantial proportion of apparently healthy individuals [7,8,9,10].

Hs-TnI concentrations were categorized using predefined sex-specific thresholds reported for the Abbott ARCHITECT STAT High Sensitive Troponin-I assay (Abbott Laboratories; REF 3P25) and supported by population-based evidence, including the Nord-Trondelag Health (HUNT) Study and recent expert summaries of hs-cTnI use in asymptomatic individuals [18,24,25]. For women, hs-TnI concentrations were categorized as low (<4 ng/L), intermediate (4–10 ng/L), or elevated (>10 ng/L); for men, the corresponding thresholds were <6 ng/L, 6–12 ng/L, and >12 ng/L. These categories were used for exploratory cross-sectional biomarker categorization and should not be interpreted as guideline-defined prognostic risk strata or as diagnostic thresholds for acute myocardial infarction.

### 2.3. Conventional Cardiovascular Assessment Methods

The Framingham Risk Score was calculated using the available dataset variables, including age, sex, cholesterol variables, systolic blood pressure and blood pressure status, smoking status, and diabetes status, and was then grouped into low, intermediate, and high categories according to the predefined dataset categories [3]. Both cholesterol/HDL and LDL/HDL ratios were reported as continuous lipid-derived indices in the descriptive analyses. For categorical concordance analyses, however, a single lipid-ratio comparator was used to avoid redundancy and inconsistent interpretation across highly related lipid indices. The predefined cholesterol/HDL-ratio category available in the dataset was therefore selected as the lipid-based categorical comparator. This variable was used only as an exploratory lipid-derived comparator and not as a substitute for validated multivariable cardiovascular risk prediction models. As an additional European-calibrated comparator, SCORE2 was calculated in eligible participants using age, sex, smoking status, systolic blood pressure, total cholesterol, and HDL cholesterol, with Italy treated as a moderate-risk European region [5,6]. SCORE2 analyses were restricted to eligible participants (*n* = 287).

Terminology was standardized throughout the manuscript: sex was used for biological male/female comparisons, high-sensitivity cardiac troponin I was used instead of high-sensitive troponin I, and hs-TnI was used as the uniform abbreviation.

### 2.4. Statistical Analysis

Continuous variables were inspected using graphical assessment and the Shapiro–Wilk test. Because hs-TnI and several clinical variables were non-normally distributed, continuous variables are reported as median [interquartile range; minimum–maximum] unless otherwise specified. Between-sex comparisons used the Mann–Whitney U test for continuous variables and the chi-square or Fisher exact test for categorical variables. Comparisons across hs-TnI categories used the Kruskal–Wallis test for continuous variables and the chi-square or Fisher exact test for categorical variables.

Spearman correlation coefficients were calculated between continuous hs-TnI and continuous clinical variables. Agreement between hs-TnI categories and comparator categories was evaluated using Cohen’s kappa and quadratic weighted kappa with bootstrap 95% confidence intervals. Multivariable linear regression was performed using log (hs-TnI + 1) as the dependent variable. A logistic regression model was also used to explore factors associated with intermediate/elevated hs-TnI categories versus the low hs-TnI category. Sensitivity analyses were performed after excluding the most extreme hs-TnI value. Supplementary concordance analyses additionally compared hs-TnI categories with SCORE2 categories in SCORE2-eligible participants. A *p*-value <0.05 was considered statistically significant.

Effect sizes were estimated as rank-biserial correlations for two-group continuous comparisons, epsilon-squared (ε^2^) for Kruskal–Wallis comparisons, and Cramér’s V for categorical comparisons. Bootstrap resampling was used to derive 95% confidence intervals. To assess whether the main findings depended on the manufacturer-based cut-points, a sensitivity analysis categorized hs-TnI using sex-specific empirical percentile ranks. Tied concentrations were retained at their average rank; the lower, middle, and upper groups were defined by percentile thresholds of ≤33.3%, >33.3% to ≤66.7%, and >66.7%, respectively. Raw agreement and quadratic weighted kappa with bootstrap 95% confidence intervals were recalculated for each comparator.

## 3. Results

### 3.1. Study Population

A total of 292 apparently healthy blood donors were included in the analysis, comprising 77 women (26.4%) and 215 men (73.6%). The age range was 40–65 years, with a median age of 49 years [IQR, 45–55]. The cohort was therefore predominantly male and middle-aged, a feature that should be considered when interpreting both hs-TnI distributions and the external validity of the findings.

The main clinical and biochemical characteristics of the study population are reported in Table 1. Men showed higher BMI, fasting glucose, triglycerides, creatinine, systolic blood pressure, cholesterol/HDL ratio, LDL/HDL ratio, and hs-TnI concentrations, whereas women showed higher HDL cholesterol and total cholesterol. These sex-related differences are consistent with the known influence of biological sex on lipid profile, renal-related laboratory variables, blood pressure, and circulating hs-TnI concentrations.

Effect-size estimates were consistent with these descriptive differences. The largest between-sex effects were observed for creatinine (rank-biserial r = 0.832, 95% CI 0.769 to 0.888), SCORE2 risk (r = 0.624, 95% CI 0.500 to 0.734), Framingham score (r = 0.613, 95% CI 0.489 to 0.717), systolic blood pressure (r = 0.546, 95% CI 0.419 to 0.670), and hs-TnI (r = 0.477, 95% CI 0.356 to 0.593). Complete effect-size estimates are reported in Supplementary Table S4A.

Both cholesterol/HDL ratio and LDL/HDL ratio were retained as continuous lipid-derived indices in the descriptive analyses. For categorical concordance analyses, however, only the predefined cholesterol/HDL-ratio category was used as the lipid-derived categorical comparator, to avoid redundancy across highly related lipid indices.

### 3.2. Distribution of hs-TnI Categories and Association with Clinical Variables

Using the predefined sex-specific hs-TnI thresholds, 253 participants (86.6%) were classified in the low hs-TnI category, 17 (5.8%) in the intermediate category, and 22 (7.5%) in the elevated category. Hs-TnI concentrations were markedly skewed, particularly in the elevated category. Median hs-TnI was 2.0 ng/L [IQR, 1.0–2.0; range, 0–5] in the low category, 9.0 ng/L [IQR, 7.0–10.0; range, 6–12] in the intermediate category, and 21.5 ng/L [IQR, 15.25–36.0; range, 11–199] in the elevated category.

The comparison of clinical and biochemical variables across predefined hs-TnI categories is shown in Table 2. Apart from hs-TnI itself, creatinine showed a small between-category difference (ε^2^ = 0.016, 95% CI 0.000 to 0.060; *p* = 0.039). All other conventional cardiovascular variables, including age, BMI, lipid parameters, cholesterol/HDL ratio, LDL/HDL ratio, CRP, systolic blood pressure, smoking status, family history, diabetes/prediabetes status, Framingham categories, and SCORE2 categories, did not differ significantly across the three hs-TnI groups. Fasting glucose showed a non-significant trend with a small effect size (ε^2^ = 0.010, 95% CI 0.000 to 0.056; *p* = 0.087). Effect sizes for the remaining continuous variables were negligible (ε^2^ ≤ 0.010), and categorical associations were small (Cramér’s V = 0.028–0.114), as detailed in Supplementary Table S4B. Similarly, SCORE2 risk percentage did not show a significant gradient across hs-TnI categories among eligible participants. Median SCORE2 risk was 3.1% [IQR, 1.9–5.2] in the low hs-TnI category, 2.5% [IQR, 1.7–2.9] in the intermediate category, and 3.4% [IQR, 2.0–5.9] in the elevated category (*p* = 0.233). This finding indicates that individuals with intermediate or elevated hs-TnI were not simply those assigned to higher risk categories by SCORE2.

Overall, Table 2 supports the interpretation that predefined hs-TnI categories identify a biomarker-defined pattern that is only partially explained by conventional cardiovascular risk variables in this selected donor cohort.

### 3.3. Correlation and Multivariable Analyses

Because hs-TnI and most continuous clinical variables were non-normally distributed, correlation analyses were performed using Spearman’s rho. Continuous hs-TnI showed weak correlations with age, BMI, fasting glucose, HDL cholesterol, cholesterol/HDL ratio, creatinine, systolic blood pressure, Framingham score, and SCORE2 risk percentage. No relevant correlation was observed with total cholesterol, triglycerides, LDL cholesterol, or CRP. The complete correlation matrix is reported in Supplementary Table S1.

In multivariable linear regression using log(hs-TnI + 1) as the dependent variable, conventional cardiovascular variables explained only a limited proportion of hs-TnI variability. In the logistic model evaluating intermediate/elevated hs-TnI categories versus the low hs-TnI category, conventional cardiovascular variables did not identify a robust independent explanatory profile. SCORE2 was not included as a covariate in the main multivariable model because it is derived from conventional risk-factor variables already considered in the analysis. Exploratory sensitivity analyses excluding the most extreme hs-TnI value did not materially change the direction of the main findings.

The adjusted linear model explained a limited proportion of log-transformed hs-TnI variability (adjusted R^2^ = 0.075). The adjusted estimate for male sex was β = 0.291 (95% CI 0.000 to 0.581), while the estimate for creatinine was β = 0.560 (95% CI −0.248 to 1.368). Smoking showed an inverse adjusted association (β = −0.257, 95% CI −0.483 to −0.032). In the logistic model, all covariate odds-ratio confidence intervals included 1. Complete multivariable estimates and 95% confidence intervals are reported in Supplementary Table S5.

These results should not be interpreted as evidence of prognostic independence, because no outcome data were available. Rather, they support the cross-sectional observation that hs-TnI does not behave as a simple surrogate of the conventional risk-factor burden captured by Framingham, cholesterol/HDL-ratio categories, or SCORE2.

### 3.4. Distribution of Cardiovascular Assessment Categories Across Methods

**Figure 1:**
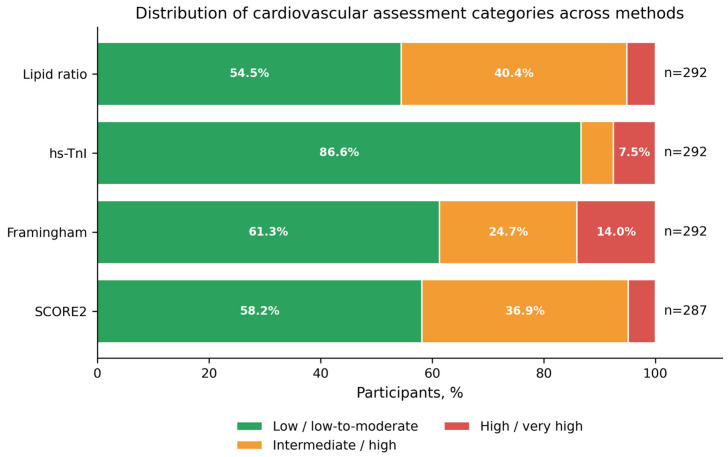
**Distribution of cardiovascular assessment categories across methods.** Stacked bars show the percentage of participants classified as low, intermediate, or high/elevated by cholesterol/HDL-ratio categories, hs-TnI categories, Framingham Risk Score categories, and SCORE2 categories. For color consistency across methods, the SCORE2 classes low-to-moderate, high, and very high are displayed using the same low/intermediate/high color palette. Absolute numbers are summarized in Supplemental Table S3; SCORE2 was available for eligible participants only (*n* = 287).

The distribution of participants across cardiovascular assessment categories differed substantially according to the method used (Supplementary Table S3 and Figure 1). Hs-TnI classified most participants as low category, with a smaller subset assigned to intermediate or elevated categories. Framingham and SCORE2 produced broader distributions across low/intermediate/high or low-to-moderate/high/very-high categories, whereas the cholesterol/HDL-ratio category showed a different lipid-driven pattern.

Hs-TnI classified 253/292 participants (86.6%) as low, 17/292 (5.8%) as intermediate, and 22/292 (7.5%) as elevated. Framingham classified 179/292 (61.3%) as low, 72/292 (24.7%) as intermediate, and 41/292 (14.0%) as high. The cholesterol/HDL-ratio category classified 159/292 (54.5%) as low, 118/292 (40.4%) as intermediate, and 15/292 (5.1%) as high. SCORE2 was calculable in 287 eligible participants; among them, 167 (58.2%) were classified as low-to-moderate risk, 106 (36.9%) as high risk, and 14 (4.9%) as very high risk.

This different distribution is central to the interpretation of the study. The hs-TnI categories did not reproduce the allocation generated by either traditional Framingham-based assessment, cholesterol/HDL-ratio categories, or the more contemporary European SCORE2 model.

### 3.5. Concordance and Discordance Between hs-TnI and Conventional Assessment Methods

**Figure 2:**
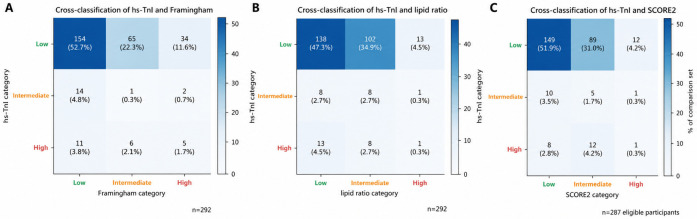
Cross-classification of hs-TnI with Framingham, cholesterol/HDL-ratio categories, and SCORE2. Composite heatmap panels summarize pairwise cross-classification between hs-TnI categories and comparator methods. (**A**) shows hs-TnI versus Framingham (*n* = 292), (**B**) shows hs-TnI versus cholesterol/HDL-ratio categories (*n* = 292), and (**C**) shows hs-TnI versus SCORE2 (*n* = 287 eligible participants). Each cell reports the number of participants and the percentage of the comparison set assigned to the corresponding category pair. Across all three comparisons, the limited concentration of observations along the diagonal illustrates low categorical agreement.

Cross-classification analyses confirmed the limited overlap between hs-TnI categories and the comparator methods. The composite heatmap in Figure 2 illustrates the pairwise distribution of hs-TnI categories against Framingham (Panel A), cholesterol/HDL-ratio categories (Panel B), and SCORE2 (Panel C). In each comparison, a large proportion of individuals classified as low by hs-TnI were distributed across higher categories by the comparator method, whereas only a minority of individuals in intermediate or elevated hs-TnI categories were concordantly assigned to higher comparator categories.

Agreement statistics were consistently low. Raw agreement was 54.8% for hs-TnI versus Framingham, 50.3% for hs-TnI versus cholesterol/HDL-ratio categories, and 54.0% for hs-TnI versus SCORE2 among eligible participants. Quadratic weighted kappa values were 0.035 for hs-TnI versus Framingham, −0.008 for hs-TnI versus cholesterol/HDL-ratio categories, and 0.076 for hs-TnI versus SCORE2. Complete agreement statistics, including Cohen’s kappa and bootstrap confidence intervals, are provided in Supplementary Table S2.

The low agreement observed across all three comparisons indicates that hs-TnI-based categorization is not redundant with conventional risk-factor-based assessment. Importantly, this discordance should not be interpreted as evidence that hs-TnI provides superior or more accurate risk assignment. In the absence of follow-up outcomes, cardiovascular events, or imaging endpoints, the observed discordance should be interpreted as a hypothesis-generating finding, consistent with the concept that hs-TnI may capture a cardiac-specific biomarker dimension not fully reflected by conventional cardiovascular risk scores.

### 3.6. Sex-Specific Distribution of Assessment Categories

**Figure 3:**
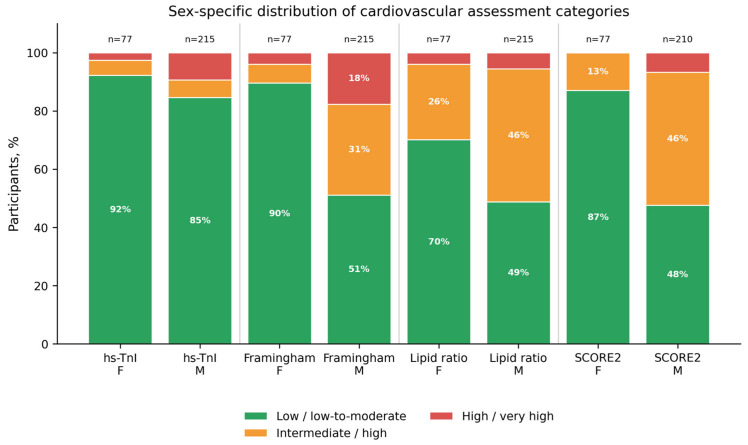
Sex-specific distribution of cardiovascular assessment categories across methods. Stacked bars display female and male category distributions for hs-TnI, Framingham, cholesterol/HDL-ratio categories, and SCORE2. The figure highlights sex-related differences in category distribution in a predominantly male cohort and includes SCORE2 only for eligible participants.

Sex-specific distributions are shown in Figure 3. The analysis confirmed the expected sex-related differences in cardiovascular assessment profiles within this predominantly male cohort. Men showed a broader distribution across higher categories for several conventional assessment methods, consistent with their higher BMI, systolic blood pressure, creatinine, triglycerides, cholesterol/HDL ratio, LDL/HDL ratio, and hs-TnI concentrations. Women were more frequently represented in lower conventional risk categories, although hs-TnI categories remained present in both sexes.

These sex-specific findings reinforce the need for sex-specific interpretation of hs-TnI concentrations and support the use of predefined sex-specific hs-TnI thresholds in the present exploratory analysis.

### 3.7. Sensitivity Analysis Using Sex-Specific Empirical hs-TnI Tertiles

The sensitivity categorization allocated 80 participants (27.4%) to the lower tertile, 105 (36.0%) to the middle tertile, and 107 (36.6%) to the upper tertile. Agreement with conventional assessment methods remained low. Raw agreement was 32.5% with Framingham, 30.1% with cholesterol/HDL-ratio categories, and 31.0% with SCORE2. Quadratic weighted kappa values were 0.045 (95% CI −0.041 to 0.134), 0.033 (95% CI −0.047 to 0.112), and 0.037 (95% CI −0.046 to 0.117), respectively. Thus, the limited categorical overlap was retained when hs-TnI was categorized independently of the manufacturer-based thresholds (Supplementary Table S6).

## 4. Discussion

This cross-sectional study in apparently healthy blood donors shows that predefined sex-specific hs-TnI categories identify a small subset of individuals with intermediate or elevated hs-TnI concentrations. These categories showed limited agreement with Framingham and cholesterol/HDL-ratio categories, and this pattern remained essentially unchanged when SCORE2 was added as a contemporary European comparator in eligible participants. Taken together, these findings suggest that hs-TnI captures a biomarker pattern that is not simply interchangeable with conventional cardiovascular assessment. This observation is consistent with the broader literature describing hs-cTnI as a cardiac-specific marker detectable in asymptomatic individuals and potentially useful for risk refinement when interpreted as an adjunct, rather than as a stand-alone screening test [11,12,13,14,15,16,17,18].

The main finding is twofold. First, even in a pre-selected donor population, 39 of 292 individuals (13.4%) fell into intermediate or elevated hs-TnI categories, indicating measurable heterogeneity in low-grade myocardial injury signals despite apparent clinical health. Second, the very low agreement with Framingham, cholesterol/HDL-ratio categories, and SCORE2 indicates that this biomarker-defined pattern is not merely a duplicate of conventional risk-factor categorization. This is the principal novelty of the study and provides a rationale for future longitudinal work, rather than immediate clinical implementation.

The findings should not be read as evidence that hs-TnI provides validated prognostic reclassification in this cohort. A more appropriate interpretation is that hs-TnI describes a biomarker pattern that only partly overlaps with risk-factor-based approaches. This observation is biologically plausible because cardiac troponin reflects myocardial injury or cardiomyocyte stress, whereas traditional scores quantify cumulative exposure to risk factors. In addition, increasing evidence suggests that inflammatory pathways and innate immune mechanisms may contribute to subclinical organ injury and cardiovascular damage, supporting the concept that biomarker-based approaches can capture biological processes not fully reflected by traditional risk-factor assessment [26]. Farmakis et al. recently summarized evidence supporting the additive value of hs-cTnI on top of established cardiovascular risk tools in asymptomatic populations, including prospective cohorts such as HUNT, BiomarCaRE, WOSCOPS, JUPITER, ARIC, and MORGAM [18]. In that conceptual framework, the additional inclusion of SCORE2 in our revised analysis is useful because it shows that the discordance is not restricted to Framingham alone. Our data do not test prognostic additive value directly; rather, they provide a real-world laboratory description of how predefined hs-TnI categories distribute within a selected donor population and how they compare with both traditional and contemporary risk-factor-based methods.

At the same time, hs-TnI was not completely detached from conventional clinical variables when examined as a continuous measure. Weak correlations were observed with systolic blood pressure, creatinine, glucose, BMI, HDL cholesterol, Framingham score, and SCORE2 risk percentage. These findings support a balanced interpretation: hs-TnI may reflect a cardiac-specific injury dimension, but its concentrations are still shaped by demographic, renal, hemodynamic, and metabolic factors.

The effect-size analysis further qualifies this finding. Across the predefined hs-TnI categories, the between-group effect was negligible for most conventional variables and small for creatinine (ε^2^ = 0.016). Thus, statistically significant correlations observed when hs-TnI was analyzed continuously did not translate into clearly separated conventional risk-factor profiles across the predefined categories. This supports partial biological overlap without suggesting that hs-TnI is independent of renal, hemodynamic, or metabolic influences.

The low kappa values between hs-TnI and conventional categories quantify the discordance shown in the heatmaps. In the absence of prospective outcomes, imaging endpoints, or intervention data, however, this discordance remains hypothesis-generating. It shows difference, not superiority, and cannot by itself demonstrate improved prediction, management benefit, or clinical utility as a screening test.

The sensitivity analysis directly addressed whether this pattern was an artifact of the selected manufacturer-based cut-points. When sex-specific empirical tertiles were used, weighted kappa remained close to zero for Framingham (0.045), cholesterol/HDL-ratio categories (0.033), and SCORE2 (0.037), with all bootstrap confidence intervals including zero. Because the tertile approach produced broader and more balanced hs-TnI groups than the predefined thresholds, the persistence of low agreement indicates that the observed cross-method differences were not solely driven by the original threshold scheme. This robustness analysis strengthens the cross-sectional conclusion of non-redundant category allocation, while still providing no evidence of prognostic superiority.

From a laboratory medicine perspective, these findings argue for careful interpretative frameworks if hs-TnI is measured outside acute care. The challenges related to analytical validation, clinical interpretation, and integration of novel biomarkers into patient management are not unique to cardiovascular medicine and have been described in other biomarker-based clinical settings [27].

Reports should make clear that detectable or modestly elevated hs-TnI in asymptomatic individuals does not diagnose acute myocardial infarction and should not automatically trigger unstructured downstream testing. Practical safeguards could include standardized interpretive comments, repeat testing only when clinically appropriate, integration with established cardiovascular risk assessment, and attention to renal function, blood pressure, recent strenuous exercise, and other non-acute causes of troponin elevation [18,19,20,21,22,28,29,30,31,32]. This guarded approach is consistent with expert recommendations that hs-cTnI should be considered as an adjunctive signal within structured prevention pathways rather than as an isolated decision rule.

The main strength of this study is the evaluation of hs-TnI in a standardized, apparently healthy donor cohort, with direct comparison of biomarker-defined categories and conventional cardiovascular assessment methods in the same individuals. The findings should nevertheless be interpreted within the boundaries of the study design. Because the analysis was cross-sectional, it could not assess incident cardiovascular events, cardiovascular mortality, heart failure, imaging endpoints, or the effect of hs-TnI-guided clinical decisions. Hs-TnI was measured at a single time point, so intra-individual biological variation and serial change were not evaluated. Although the revised analysis included several relevant variables, including systolic blood pressure, creatinine, diabetes/prediabetes status, smoking, BMI, family history, and the variables required for SCORE2, other potentially informative determinants were not available, such as medication use, physical activity, electrocardiographic or echocardiographic findings, NT-proBNP, ApoB, Lipoprotein(a), and HbA1c. Renal status was represented by creatinine rather than by a fully reported eGFR-based analysis. In addition, the selected donor population, the predominance of men, and the age range of the cohort limit generalizability to women, older adults, patients with comorbidities, and unselected primary-care populations. Finally, the predefined hs-TnI thresholds used in this study were applied for exploratory biomarker categorization; they should not be interpreted as guideline-defined prognostic strata or as evidence that hs-TnI-guided preventive interventions improve outcomes. SCORE2 was available only for eligible participants and is therefore presented as a supplementary comparator. These limitations align with open issues highlighted in recent reviews, including the need for prospective outcome-based studies, serial hs-cTnI assessment, and clearly defined clinical pathways before population-level implementation [18].

First-time versus repeat donor status was not recorded in the analytical dataset, which prevents assessment of potential differences related to donation history. Moreover, the tertile sensitivity analysis was data-driven and affected by tied low hs-TnI concentrations; empirical average ranks were therefore used to retain identical concentrations within the same category. These considerations do not alter the principal comparison but should be considered when interpreting external validity and the sensitivity results.

## 5. Conclusions

In apparently healthy blood donors, predefined sex-specific hs-TnI categories showed limited concordance with Framingham and cholesterol/HDL-ratio categories, and the same low concordance was observed when SCORE2 was examined in eligible participants. The main contribution of this study is the identification of a distinct cross-sectional hs-TnI biomarker phenotype within a selected apparently healthy population, rather than the validation of a new prognostic risk score. These findings are consistent with the concept that hs-TnI may describe low-grade myocardial injury not fully captured by conventional risk-factor categorization, but prospective studies with clinical outcomes are required to determine whether this biomarker pattern improves cardiovascular risk prediction or supports actionable prevention pathways [18]. The persistence of low agreement in the sex-specific empirical-tertile analysis indicates that this conclusion was not dependent on a single hs-TnI threshold scheme.

## Figures and Tables

**Table 1:** Clinical characteristics of analyzed subjects according to sex. Effect sizes and bootstrap 95% confidence intervals for these comparisons are reported in Supplementary Table S4A. Continuous variables are reported as median [interquartile range; min–max]. Categorical variables are reported as *n* (%), unless otherwise specified. The BMI category row reports the distribution across normal weight/pre-obese/obese categories as *n* (%) for each category. *p* values refer to Mann–Whitney U tests for continuous variables and chi-square or Fisher exact tests for categorical variables, as appropriate. * SCORE2 risk and categories are reported only among SCORE2-eligible participants.

Variable	Overall (*n* = 292)	Female (*n* = 77)	Male (*n* = 215)	*p* Value
Age, years, median [IQR; min–max]	49.00 [45.00, 55.00; 40.00–65.00]	49.00 [45.00, 54.00; 40.00–64.00]	50.00 [45.00, 55.00; 40.00–65.00]	0.270
BMI, kg/m^2^, median [IQR; min–max]	25.87 [24.00, 28.09; 18.70–38.97]	24.58 [22.32, 27.18; 19.49–36.33]	26.23 [24.69, 28.52; 18.70–38.97]	<0.001
Fasting glucose, mg/dL, median [IQR; min–max]	91.00 [85.00, 97.00; 64.00–150.00]	86.00 [83.00, 93.00; 69.00–122.00]	92.00 [87.00, 98.00; 64.00–150.00]	<0.001
Total cholesterol, mg/dL, median [IQR; min–max]	177.60 [156.88, 203.60; 103.33–326.04]	190.26 [163.21, 208.79; 120.25–326.04]	174.10 [155.84, 202.22; 103.33–282.00]	0.041
Triglycerides, mg/dL, median [IQR; min–max]	79.78 [59.54, 108.28; 32.72–615.25]	66.98 [54.72, 99.43; 32.72–615.25]	83.94 [63.49, 110.15; 33.24–543.00]	0.008
LDL cholesterol, mg/dL, median [IQR; min–max]	131.00 [110.00, 152.00; 37.00–260.00]	125.00 [108.00, 145.00; 37.00–260.00]	131.74 [111.00, 153.50; 67.00–229.00]	0.114
HDL cholesterol, mg/dL, median [IQR; min–max]	54.00 [46.00, 63.00; 28.00–108.00]	63.00 [57.00, 72.00; 28.00–108.00]	50.00 [44.00, 59.00; 30.00–90.00]	<0.001
Cholesterol/HDL ratio, median [IQR; min–max]	3.29 [2.73, 3.88; 1.32–6.48]	2.83 [2.43, 3.46; 1.32–6.02]	3.48 [2.92, 3.97; 1.93–6.48]	<0.001
LDL/HDL ratio, median [IQR; min–max]	2.40 [1.84, 3.06; 0.72–5.38]	1.98 [1.53, 2.48; 0.72–4.80]	2.62 [2.02, 3.15; 0.83–5.38]	<0.001
hs-TnI, ng/L, median [IQR; min–max]	2.00 [1.00, 3.00; 0.00–199.00]	1.00 [1.00, 2.00; 0.00–37.00]	2.00 [1.00, 3.00; 0.00–199.00]	<0.001
CRP, mg/dL, median [IQR; min–max]	0.12 [0.06, 0.22; 0.02–8.67]	0.10 [0.07, 0.22; 0.02–1.02]	0.12 [0.06, 0.21; 0.02–8.67]	0.866
Creatinine, mg/dL, median [IQR; min–max]	0.87 [0.75, 0.97; 0.52–1.30]	0.71 [0.64, 0.77; 0.52–0.89]	0.93 [0.84, 1.01; 0.57–1.30]	<0.001
Systolic blood pressure, mmHg, median [IQR; min–max]	119.00 [117.00, 132.00; 113.00–174.00]	117.00 [115.00, 119.00; 113.00–156.00]	127.00 [118.00, 138.50; 114.00–174.00]	<0.001
Framingham score, %, median [IQR; min–max]	8.20 [5.10, 14.22; 1.30–60.60]	4.70 [3.10, 6.80; 1.30–19.60]	9.70 [6.75, 15.90; 2.20–60.60]	<0.001
SCORE2 risk, %, median [IQR; min–max] *	3.10 [1.90, 5.20; 0.50–15.00]	1.50 [0.90, 2.80; 0.50–7.20]	3.75 [2.30, 5.78; 0.90–15.00]	<0.001
SCORE2 category distribution (low-to-moderate/high/very high), *n* (%)	167 (58.2)/106 (36.9)/14 (4.9)	67 (87.0)/10 (13.0)/0 (0.0)	100 (47.6)/96 (45.7)/14 (6.7)	<0.001
Smokers, *n* (%)	72 (24.7)	20 (26.0)	52 (24.2)	0.874
Hypertension/borderline BP, *n* (%)	56 (19.2)	8 (10.4)	48 (22.3)	0.035
Family history, *n* (%)	99 (33.9)	24 (31.2)	75 (34.9)	0.652
Prediabetes/diabetes, *n* (%)	22 (7.5)	7 (9.1)	15 (7.0)	0.154
BMI category distribution (normal/pre-obese/obese), *n* (%) for each category	106/144/42	42/25/10	64/119/32	<0.001

**Table 2:** Clinical correlates of analyzed subjects according to predefined hs-TnI categories. Effect sizes and bootstrap 95% confidence intervals for these comparisons are reported in Supplementary Table S4B. Continuous variables are reported as median [interquartile range; min–max]. Categorical variables are reported as *n* (%). *p* values refer to Kruskal–Wallis tests for continuous variables and chi-square or Fisher exact tests for categorical variables, as appropriate. * SCORE2 risk and categories are reported only among SCORE2-eligible participants.

Variable	Low hs-TnI (*n* = 253)	Intermediate hs-TnI (*n* = 17)	Elevated hs-TnI (*n* = 22)	*p* Value
Age, years, median [IQR; min–max]	49.00 [45.00, 55.00; 40.00–65.00]	49.00 [45.00, 52.00; 40.00–64.00]	49.50 [45.00, 57.50; 40.00–64.00]	0.628
BMI, kg/m^2^, median [IQR; min–max]	25.83 [24.06, 28.09; 18.70–38.97]	25.56 [22.90, 29.39; 22.04–33.25]	26.61 [24.76, 28.02; 19.79–33.95]	0.791
Fasting glucose, mg/dL, median [IQR; min–max]	91.00 [85.00, 96.00; 64.00–137.00]	87.00 [85.00, 95.00; 77.00–126.00]	95.50 [88.50, 100.00; 80.00–150.00]	0.087
Total cholesterol, mg/dL, median [IQR; min–max]	176.86 [156.52, 204.01; 103.33–326.04]	178.09 [159.52, 194.80; 137.66–257.33]	181.50 [159.72, 210.18; 137.48–282.00]	0.854
Triglycerides, mg/dL, median [IQR; min–max]	82.17 [61.91, 108.98; 32.72–615.25]	59.54 [51.64, 82.38; 45.08–220.47]	69.00 [56.26, 88.00; 33.24–543.00]	0.090
LDL cholesterol, mg/dL, median [IQR; min–max]	131.00 [111.00, 153.00; 37.00–260.00]	135.00 [109.14, 139.00; 76.48–172.00]	124.50 [103.25, 146.25; 81.19–192.00]	0.685
HDL cholesterol, mg/dL, median [IQR; min–max]	54.00 [46.00, 63.00; 28.00–108.00]	49.00 [45.00, 59.00; 33.00–82.00]	54.50 [46.25, 64.25; 31.00–80.00]	0.741
Cholesterol/HDL ratio, median [IQR; min–max]	3.28 [2.73, 3.89; 1.32–6.48]	3.56 [3.07, 3.86; 2.18–5.36]	3.23 [2.68, 3.78; 2.37–6.27]	0.753
LDL/HDL ratio, median [IQR; min–max]	2.40 [1.84, 3.07; 0.72–5.38]	2.38 [1.88, 3.10; 1.20–3.71]	2.39 [1.77, 2.91; 1.40–4.27]	0.852
hs-TnI, ng/L, median [IQR; min–max]	2.00 [1.00, 2.00; 0.00–5.00]	9.00 [7.00, 10.00; 6.00–12.00]	21.50 [15.25, 36.00; 11.00–199.00]	<0.001
CRP, mg/dL, median [IQR; min–max]	0.12 [0.07, 0.22; 0.02–8.67]	0.14 [0.05, 0.22; 0.02–0.39]	0.11 [0.06, 0.21; 0.03–0.85]	0.962
Creatinine, mg/dL, median [IQR; min–max]	0.86 [0.74, 0.96; 0.54–1.30]	0.88 [0.82, 0.95; 0.61–1.01]	0.94 [0.88, 1.04; 0.52–1.14]	0.039
Systolic blood pressure, mmHg, median [IQR; min–max]	119.00 [117.00, 131.00; 113.00–174.00]	118.00 [116.00, 139.00; 115.00–174.00]	127.00 [118.00, 138.50; 115.00–168.00]	0.344
Framingham score, %, median [IQR; min–max]	8.30 [5.10, 13.80; 1.30–60.60]	6.30 [5.20, 8.60; 3.30–36.00]	10.15 [6.47, 16.38; 2.60–30.00]	0.314
SCORE2 risk, %, median [IQR; min–max] *	3.10 [1.90, 5.17; 0.50–15.00]	2.50 [1.73, 2.85; 0.80–12.00]	3.40 [2.00, 5.90; 0.90–11.00]	0.233
SCORE2 category distribution (low-to-moderate/high/very high), *n* (%) among eligible *	149 (59.6)/89 (35.6)/12 (4.8)	10 (62.5)/5 (31.2)/1 (6.2)	8 (38.1)/12 (57.1)/1 (4.8)	0.374
Male sex, *n* (%)	182 (71.9)	13 (76.5)	20 (90.9)	0.148
Smokers, *n* (%)	64 (25.3)	3 (17.6)	5 (22.7)	0.760
Hypertension/borderline BP, *n* (%)	47 (18.6)	4 (23.5)	5 (22.7)	0.800
Family history, *n* (%)	87 (34.4)	5 (29.4)	7 (31.8)	0.895
Prediabetes/diabetes, *n* (%)	18 (7.1)	2 (11.8)	2 (9.1)	0.513

## Data Availability

The original contributions presented in this study are included in the article/Supplementary Materials. Further inquiries can be directed to the corresponding author. Supplementary Data Table S1 accompanies the revised submission and flags SCORE2 eligibility while providing participant-level SCORE2 results for all analyzed subjects.

## Supplementary Materials

The following supporting information can be downloaded at: https://www.mdpi.com/article/10.3390/diagnostics16142203/s1, Table S1: Spearman correlations between continuous hs-TnI concentrations and continuous clinical variables. Table S2: Agreement between hs-TnI and conventional cardiovascular assessment methods. Table S3: Distribution of categories across hs-TnI, Framingham, cholesterol/HDL-ratio, and SCORE2 methods. Table S4A: Effect sizes for comparisons by sex. Table S4B: Effect sizes for comparisons across predefined hs-TnI categories. Table S5: Multivariable model estimates with 95% confidence intervals. Table S6: Sensitivity analysis using sex-specific empirical hs-TnI tertiles.

## Abbreviations

hs-TnI, high-sensitivity cardiac troponin I; hs-cTnI, high-sensitivity cardiac troponin I; BMI, body mass index; LDL, low-density lipoprotein; HDL, high-density lipoprotein; CRP, C-reactive protein; SBP, systolic blood pressure; BP, blood pressure; SCORE2, Systematic Coronary Risk Evaluation 2; CI, confidence interval; IQR, interquartile range; OR, odds ratio; β, regression coefficient; R^2^, coefficient of determination; ρ, Spearman’s rank correlation coefficient; W, Shapiro–Wilk statistic; ε^2^, epsilon-squared; n, number; *p*, *p* value; M, male; F, female.
