# Supplementary material for: High-Sensitivity Cardiac Troponin I in Apparently Healthy Blood Donors: Cross-Sectional Distribution and Discordance with Conventional Cardiovascular Risk Assessment Methods
Source: Diagnostics (Basel). 2026 Jul 15;16(14):2203. doi: 10.3390/diagnostics16142203 (PMC13407599; doi:10.3390/diagnostics16142203)
Supplement: Supplementary file 1 [file diagnostics-16-02203-s001.zip › diagnostics-4422944-supplementary.pdf]

Supplementary Table S1

Spearman correlations between continuous hs-TnI concentrations and continuous clinical variables.

n indicates the number of subjects with available data for each variable. Spearman rho (ρ) quantifies the rank-based correlation with continuous hs-TnI concentrations. p values are two-sided. *SCORE2 risk percentage was analyzed only among SCORE2-eligible participants.

**Variable**	**n with available data**	**Spearman rho (ρ)**	**p value**
Age, years	292	0.120	0.040
BMI, kg/m2	292	0.151	0.010
Fasting glucose, mg/dL	292	0.185	0.001
Total cholesterol, mg/dL	292	0.017	0.770
Triglycerides, mg/dL	285	-0.047	0.434
LDL cholesterol, mg/dL	292	0.026	0.661
HDL cholesterol, mg/dL	292	-0.165	0.005
Cholesterol/HDL ratio	292	0.126	0.031
LDL/HDL ratio	292	0.109	0.062
CRP, mg/dL	269	0.057	0.354
Creatinine, mg/dL	290	0.332	<0.001
Systolic blood pressure, mmHg	292	0.242	<0.001
Framingham score, %	292	0.228	<0.001
SCORE2 risk, %*	287	0.192	0.001

Supplementary Table S2

Agreement between hs-TnI and conventional cardiovascular assessment methods.

Raw agreement indicates the percentage of participants assigned to the same category by hs-TnI and the comparator method. Quadratic weighted kappa was calculated to account for ordered categories; 95% confidence intervals were derived by bootstrap resampling. The cholesterol/HDL-ratio category was used as the single lipid-derived categorical comparator; LDL/HDL ratio is reported descriptively and in correlation analyses as a continuous index. For SCORE2, n refers to eligible participants only.

Comparator method	n compared	Raw agreement, %	Quadratic weighted kappa	Bootstrap 95% CI
Framingham	292	54.8	0.035	-0.076 to 0.147
Cholesterol/HDL-ratio category	292	50.3	-0.008	-0.100 to 0.095
SCORE2	287	54.0	0.076	-0.020 to 0.179

Supplementary Table S3. Distribution of categories across hs-TnI, Framingham, cholesterol/HDL-ratio, and SCORE2 methods.

Values are reported as n (%). Percentages are method-specific. Cholesterol/HDL and LDL/HDL ratios are reported as continuous variables in the descriptive tables. For categorical concordance analyses, the cholesterol/HDL-ratio category was used as the single lipid-derived comparator to avoid redundancy across related lipid indices. This category should not be interpreted as a validated multivariable cardiovascular risk score. SCORE2 was calculated in eligible participants only (n = 287).

Method	n	Low / low-to-moderate, n (%)	Intermediate / high, n (%)	High / very high, n (%)
hs-TnI	292	253 (86.6)	17 (5.8)	22 (7.5)

Framingham	292	179 (61.3)	72 (24.7)	41 (14.0)
Cholesterol/HDL-ratio category	292	159 (54.5)	118 (40.4)	15 (5.1)
SCORE2	287	167 (58.2)	106 (36.9)	14 (4.9)

Supplementary Table S4A. Effect sizes for comparisons by sex.

Rank-biserial r is positive when the distribution is higher in men. Cramér's V is a magnitude-only association measure. Confidence intervals were obtained by bootstrap resampling.

Variable	Effect size	Bootstrap 95% CI	Metric
Age, years	0.085	-0.051 to 0.227	rank-biserial r (M > F positive)
BMI, kg/m²	0.294	0.132 to 0.436	rank-biserial r (M > F positive)
Fasting glucose, mg/dL	0.289	0.136 to 0.438	rank-biserial r (M > F positive)
Total cholesterol, mg/dL	-0.157	-0.301 to -0.003	rank-biserial r (M > F positive)
Triglycerides, mg/dL	0.205	0.049 to 0.353	rank-biserial r (M > F positive)
LDL cholesterol, mg/dL	0.122	-0.033 to 0.278	rank-biserial r (M > F positive)
HDL cholesterol, mg/dL	-0.563	-0.674 to -0.437	rank-biserial r (M > F positive)
Cholesterol/HDL ratio	0.360	0.198 to 0.501	rank-biserial r (M > F positive)
LDL/HDL ratio	0.422	0.283 to 0.548	rank-biserial r (M > F positive)
hs-TnI, ng/L	0.477	0.356 to 0.593	rank-biserial r (M > F positive)
CRP, mg/dL	-0.014	-0.179 to 0.127	rank-biserial r (M > F positive)
Creatinine, mg/dL	0.832	0.769 to 0.888	rank-biserial r (M > F positive)

Variable	Effect size	Bootstrap 95% CI	Metric
Systolic blood pressure, mmHg	0.546	0.419 to 0.670	rank-biserial r (M > F positive)
Framingham score, %	0.613	0.489 to 0.717	rank-biserial r (M > F positive)
SCORE2 risk, %	0.624	0.500 to 0.734	rank-biserial r (M > F positive)
SCORE2 category distribution	0.357	0.262 to 0.446	Cramér's V
Smokers	0.018	0.001 to 0.143	Cramér's V
Hypertension/borderline BP	0.134	0.026 to 0.230	Cramér's V
Family history	0.035	0.002 to 0.146	Cramér's V
Prediabetes/diabetes	0.035	0.003 to 0.158	Cramér's V
BMI category distribution	0.232	0.128 to 0.351	Cramér's V

Supplementary Table S4B. Effect sizes for comparisons across predefined hs-TnI categories. Epsilon-squared (ε^2) is reported for continuous variables and Cramér's V for categorical variables. Confidence intervals were obtained by bootstrap resampling.

Variable	Effect size	Bootstrap 95% CI	Metric
Age, years	0.000	0.000 to 0.032	Kruskal-Wallis ε^2
BMI, kg/m²	0.000	0.000 to 0.025	Kruskal-Wallis ε^2
Fasting glucose, mg/dL	0.010	0.000 to 0.056	Kruskal-Wallis ε^2
Total cholesterol, mg/dL	0.000	0.000 to 0.023	Kruskal-Wallis ε^2
Triglycerides, mg/dL	0.010	0.000 to 0.061	Kruskal-Wallis ε^2
LDL cholesterol, mg/dL	0.000	0.000 to 0.024	Kruskal-Wallis ε^2
HDL cholesterol, mg/dL	0.000	0.000 to 0.024	Kruskal-Wallis ε^2
Cholesterol/HDL ratio	0.000	0.000 to 0.021	Kruskal-Wallis ε^2
LDL/HDL ratio	0.000	0.000 to 0.020	Kruskal-Wallis ε^2
hs-TnI, ng/L	0.368	0.363 to 0.376	Kruskal-Wallis ε^2
CRP, mg/dL	0.000	0.000 to 0.026	Kruskal-Wallis ε^2

Variable	Effect size	Bootstrap 95% CI	Metric
Creatinine, mg/dL	0.016	0.000 to 0.060	Kruskal-Wallis ε^2
Systolic blood pressure, mmHg	0.000	0.000 to 0.041	Kruskal-Wallis ε^2
Framingham score, %	0.001	0.000 to 0.035	Kruskal-Wallis ε^2
SCORE2 risk, %	0.003	0.000 to 0.039	Kruskal-Wallis ε^2
SCORE2 category distribution	0.086	0.046 to 0.189	Cramér's V
Male sex	0.114	0.043 to 0.201	Cramér's V
Smokers	0.043	0.015 to 0.162	Cramér's V
Hypertension/borderline BP	0.039	0.016 to 0.195	Cramér's V
Family history	0.028	0.016 to 0.162	Cramér's V
Prediabetes/diabetes	0.044	0.017 to 0.200	Cramér's V

Supplementary Table S5. Multivariable model estimates with 95% confidence intervals.

Model	Term	Estimate/OR	95% CI	p value	Note
Linear: log(hs-TnI+1)	const	-0.620	-2.120 to 0.880	0.416	Adj R2=0.075
Linear: log(hs-TnI+1)	Age_years	0	-0.017 to 0.016	0.958	Adj R2=0.075
Linear: log(hs-TnI+1)	SexM	0.291	0.000 to 0.581	0.050	Adj R2=0.075
Linear: log(hs-TnI+1)	BMI	-0.008	-0.038 to 0.023	0.623	Adj R2=0.075
Linear: log(hs-TnI+1)	Smoking_yes	-0.257	-0.483 to -0.032	0.026	Adj R2=0.075
Linear: log(hs-TnI+1)	Glucose	0.011	-0.001 to 0.024	0.081	Adj R2=0.075
Linear: log(hs-TnI+1)	ratio	0.001	-0.115 to 0.116	0.991	Adj R2=0.075
Linear: log(hs-TnI+1)	CRP	0.026	-0.149 to 0.200	0.774	Adj R2=0.075
Linear: log(hs-TnI+1)	Creatinine	0.560	-0.248 to 1.368	0.173	Adj R2=0.075
Linear: log(hs-TnI+1)	SBP	0.003	-0.004 to 0.010	0.380	Adj R2=0.075
Linear: log(hs-TnI+1)	Family_yes	-0.093	-0.303 to 0.118	0.386	Adj R2=0.075
Linear: log(hs-TnI+1)	Diab_abn	-0.142	-0.660 to 0.375	0.589	Adj R2=0.075

Model	Term	Estimate/OR	95% CI	p value	Note
Logistic: intermediate/elevated vs low	const	0.007	0.000 to 2.161	0.090	OR
Logistic: intermediate/elevated vs low	Age_years	0.966	0.906 to 1.029	0.284	OR
Logistic: intermediate/elevated vs low	Sex M	1.110	0.332 to 3.707	0.865	OR
Logistic: intermediate/elevated vs low	BMI	0.999	0.890 to 1.121	0.984	OR
Logistic: intermediate/elevated vs low	Smoking_yes	0.654	0.260 to 1.642	0.366	OR
Logistic: intermediate/elevated vs low	Glucose	1.030	0.983 to 1.079	0.220	OR
Logistic: intermediate/elevated vs low	ratio	0.888	0.569 to 1.387	0.602	OR
Logistic: intermediate/elevated vs low	CRP	0.736	0.173 to 3.123	0.677	OR
Logistic: intermediate/elevated vs low	Creatinine	5.799	0.290 to 115.972	0.250	OR
Logistic: intermediate/elevated vs low	SBP	1.009	0.984 to 1.035	0.466	OR
Logistic: intermediate/elevated vs low	Family_yes	0.672	0.293 to 1.543	0.349	OR
Logistic: intermediate/elevated vs low	Diab_abn	0.676	0.104 to 4.399	0.682	OR

Diab_abn: abnormal diabetes/prediabetes status; const: regression model contant; Family_yes = positive family history; Smoking_yes: current smoking status.

Supplementary Table S6. Sensitivity analysis using sex-specific empirical hs-TnI tertiles.

Tied hs-TnI concentrations were retained at their average sex-specific empirical percentile rank.

The resulting category counts were 80 lower, 105 middle, and 107 upper tertile.

Comparator	n	Raw agreement, %	Quadratic weighted kappa	Bootstrap 95% CI
Framingham	292	32.5	0.045	-0.041 to 0.134
Cholesterol/HDL-ratio category	292	30.1	0.033	-0.047 to 0.112
SCORE2	287	31.0	0.037	-0.046 to 0.117
